# Durable Disease Control With Nivolumab in Malignant Epithelioid Angiomyolipoma: A Case Report

**DOI:** 10.1002/iju5.70170

**Published:** 2026-03-18

**Authors:** Hiroki Shimoda, Shohei Fukuda, Daiki Yafuso, Kouhei Yamamoto, Yudai Ishikawa, Hiroshi Fukushima, Yuma Waseda, Hajime Tanaka, Soichiro Yoshida, Yasuhisa Fujii

**Affiliations:** ^1^ Department of Urology Institute of Science Tokyo Tokyo Japan; ^2^ Department of Human Pathology Institute of Science Tokyo Tokyo Japan

**Keywords:** CD8, epithelioid angiomyolipoma, immune checkpoint inhibitor, perivascular epithelioid cell tumors, programmed death ligand 1

## Abstract

**Introduction:**

Epithelioid angiomyolipoma is a subtype of perivascular epithelioid cell tumors that may exhibit malignant behavior. While mammalian target of rapamycin inhibitors are commonly used for systemic treatment, the efficacy of immune checkpoint inhibitors remains unclear.

**Case Presentation:**

A 58‐year‐old man was diagnosed with epithelioid angiomyolipoma after partial nephrectomy of a right renal mass. The tumor recurred locally, and multiple lung metastases developed despite surgical resection. Nivolumab was initiated after disease progression on everolimus, resulting in durable disease control for over 3 years. Immunohistochemistry revealed high PD‐L1 expression and CD8+ T‐cell infiltration within the primary tumor.

**Conclusion:**

Immune checkpoint inhibitors may be effective treatment options for malignant epithelioid angiomyolipoma with PD‐L1 expression and CD8+ T‐cell infiltration.

## Keynote Message

Herein, we report a case of malignant epithelioid angiomyolipoma in which durable disease control was achieved with nivolumab. Histopathological analysis revealed high programmed death‐ligand 1 expression and CD8+ T‐cell infiltration. These findings suggest that immune checkpoint inhibitors may be effective treatment options for malignant epithelioid angiomyolipoma with PD‐L1 expression and CD8+ T‐cell infiltration.

**Abbreviations**
AML: angiomyolipoma
CT: computed tomography
EAML: epithelioid angiomyolipoma
ICI: immune checkpoint inhibitor
IHC: immunohistochemistry
IVC: inferior vena cava
MSI: microsatellite instability
mTOR: mammalian target of rapamycin
ORR: objective response rate
PD‐1: programmed cell death 1
PD‐L1: programmed death‐ligand 1
PEComa: perivascular epithelioid cell tumor
SMA: smooth muscle actin
TMB: tumor mutation burden

## 1 Introduction

Epithelioid angiomyolipoma (EAML), a rare variant of angiomyolipoma (AML), is classified as a mesenchymal neoplasm belonging to the perivascular epithelioid cell tumor (PEComa) family [1]. Approximately 26%–48% of EAMLs exhibit malignant clinical behavior [2, 3]. Because PEComas frequently harbor *TSC1* or *TSC2* mutations, mammalian target of rapamycin (mTOR) inhibitors are commonly used in aggressive cases [1, 4, 5, 6, 7]. However, the efficacy of immune checkpoint inhibitors (ICIs) in malignant PEComas, including EAMLs, remains unclear, and reports of successful treatment are extremely limited.

## 2 Case Presentation

**FIGURE 1:**
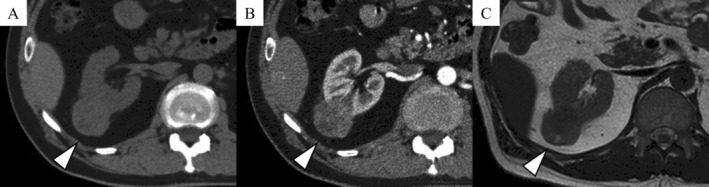
Computed tomography (A: Non‐contrast; B: Contrast‐enhanced) and magnetic resonance imaging (C: T2‐weighted imaging) reveal a 35‐mm tumor in the right kidney (arrows).

A 58‐year‐old asymptomatic man was referred to our hospital with an incidental right renal mass. He had no notable family history, including tuberous sclerosis complex. Computed tomography (CT) revealed a 35‐mm right renal tumor with slightly higher attenuation than the renal parenchyma and weak enhancement on contrast‐enhanced imaging. Magnetic resonance imaging revealed the tumor was hypointense on T2‐weighted imaging, hyperintense on diffusion‐weighted imaging, and contained minimal intratumoral fat (Figure 1). Based on these findings, differential diagnoses included papillary renal cell carcinoma and fat‐poor AML, and a partial nephrectomy was performed.

**FIGURE 2:**
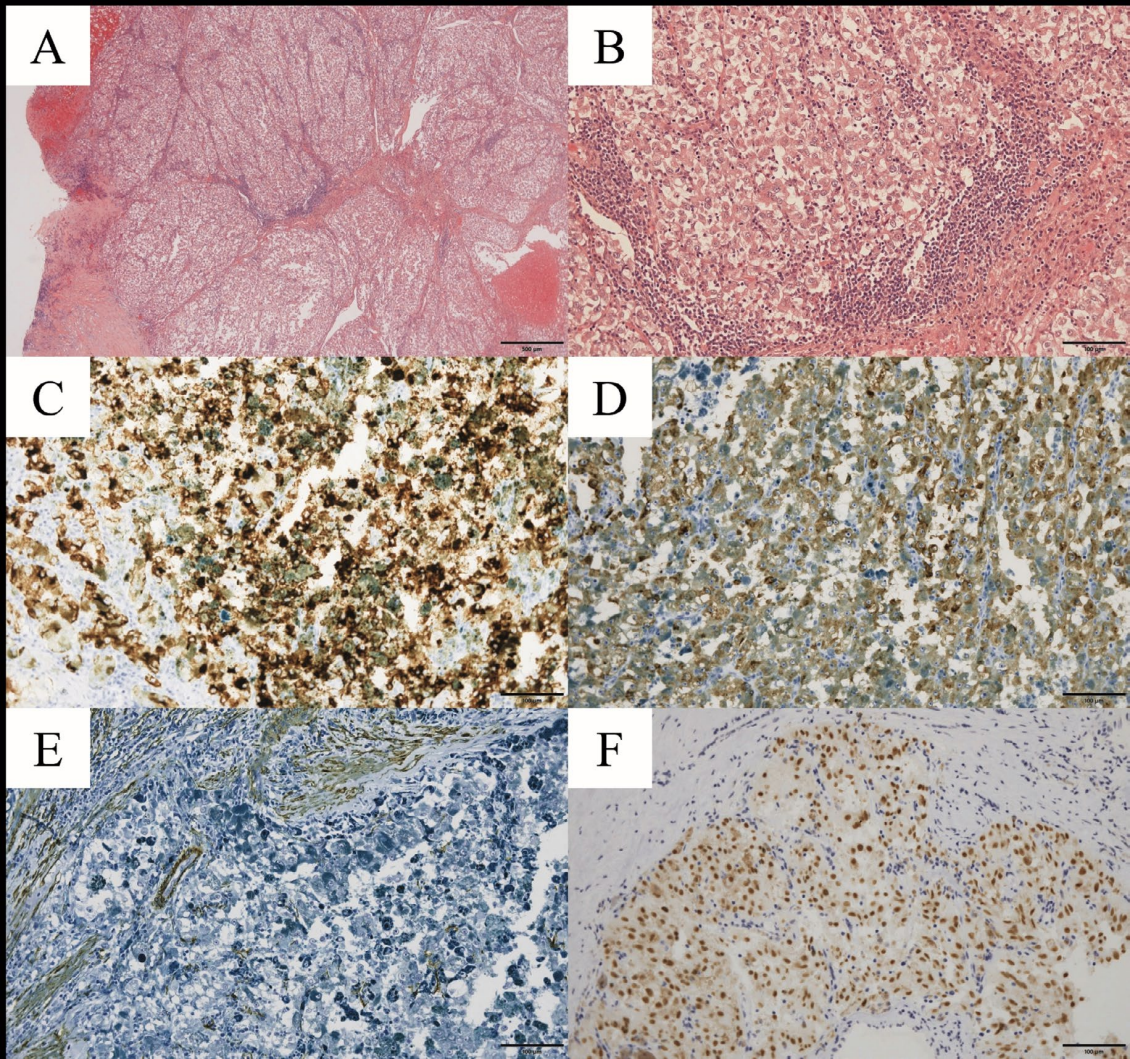
(A–E) Histopathological findings of the partial nephrectomy specimen. (A) Hematoxylin and eosin (H&E) staining, ×40; (B) H&E staining, ×200; (C) HMB‐45 immunohistochemistry; (D) Melan‐A immunohistochemistry; (E) smooth muscle actin immunohistochemistry, shown as a representative negative immunohistochemical stain; and (F) TFE3 immunohistochemistry of the nephrectomy specimen.

Grossly, the tumor measured 35 × 35 mm and appeared as a black multinodular mass with negative surgical margins. Histologically, the tumor lacked adipose and smooth muscle components and consisted of nests of epithelioid tumor cells with prominent nucleoli, large nuclei, and eosinophilic vacuolated cytoplasm. Immunohistochemistry (IHC) results were positive for HMB‐45 and Melan A and negative for S100 and SOX‐10. Additionally, negative results were observed for cytokeratin AE1/3, epithelial membrane antigen, alpha‐smooth muscle actin (SMA) (Figure 2), CD10, and c‐kit. These findings confirmed the pathological diagnosis of EAML.

Eight months after the initial surgery, CT revealed a locally recurrent tumor extending from the partial nephrectomy site to the inferior vena cava (IVC), involving para‐aortic lymph node metastasis. The patient underwent radical nephrectomy with IVC thrombectomy and lymph node dissection. Histopathological evaluation confirmed recurrent EAML with an IVC tumor thrombus and para‐aortic lymph node metastasis. Notably, IHC demonstrated positive nuclear staining for TFE3 (Figure 2), whereas fluorescence in situ hybridization (FISH) did not detect *TFE3* gene rearrangement.

**FIGURE 3:**
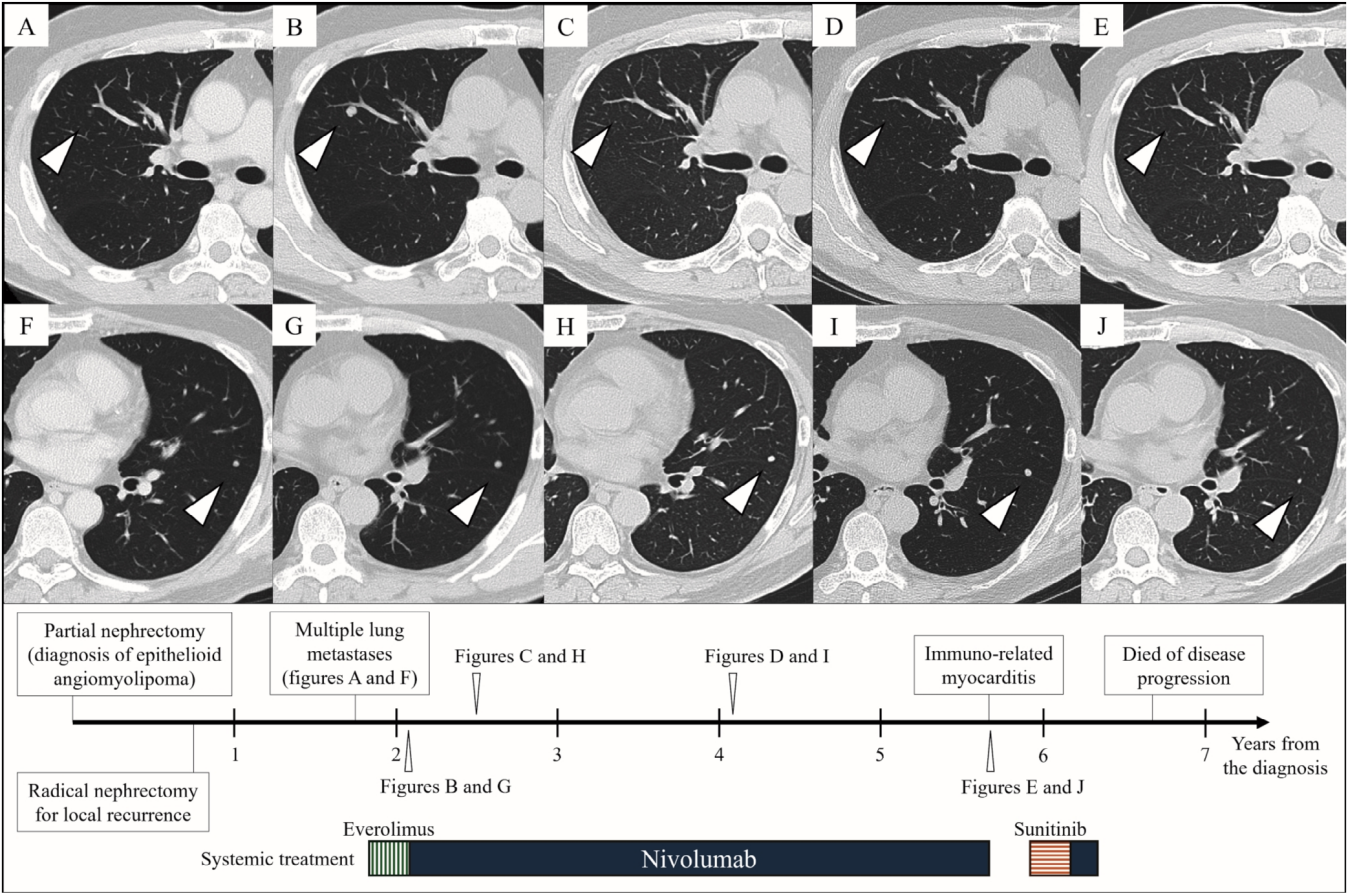
Representative changes in lung metastatic lesions and the treatment timeline following systemic therapy. Panels (A–J) show the same lesions, respectively. (A, F) Before systemic treatment; (B, G) 3 months after initiation of everolimus; (C, H) 5 months after initiation of nivolumab; (D, I) 2 years after initiation of nivolumab; (E, J) 3 years and 5 months after initiation of nivolumab (arrows).

**FIGURE 4:**
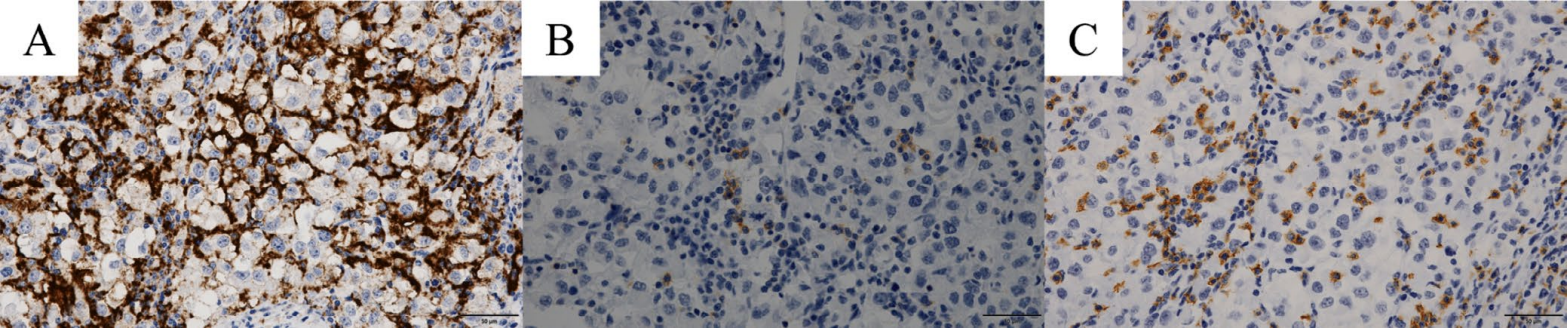
PD‐L1 (A), PD‐1 (B), and CD8 (C) immunohistochemistry of the partial nephrectomy specimen.

Ten months after the second surgery, CT identified multiple lung metastases, and everolimus was initiated. Next‐generation sequencing identified pathogenic mutations in *SDHD*, *CDKN2A/B*, and *RBM10* as well as a *TSC1* variant of uncertain significance. Microsatellite instability (MSI) was stable, and the tumor mutation burden (TMB) was low at four mutations per megabase (Muts/Mb). After 3 months, CT showed disease progression, and nivolumab was initiated. The lung metastases exhibited heterogeneous responses to nivolumab, with some lesions showing complete remission, while others demonstrated limited shrinkage, consistent with stable disease. Therefore, overall disease control was classified as stable disease according to RECIST version 1.1 (Figure 3). Disease control was maintained for 3 years and 5 months until nivolumab was discontinued because of immune‐related myocarditis. Subsequently, the disease progressed rapidly with multiple liver metastases. Despite subsequent treatment with sunitinib and nivolumab rechallenge, no response was achieved, and the patient died of disease progression 6 years and 8 months after EAML was diagnosed. Retrospective IHC of the initial tumor specimen revealed high PD‐L1 expression, CD8+ T‐cell infiltration, and a few PD‐1–positive cells (Figure 4).

## 3 Discussion

Although no standard systemic treatment strategy has been established for malignant EAML, mTOR inhibitors are frequently used in metastatic cases [1, 4, 5, 6, 7]. Guo et al. reported that 5 of 7 patients with malignant EAML achieved a clinical response to everolimus [6], while Sanfilippo et al. demonstrated an objective response rate (ORR) of 41% in 40 patients with malignant PEComa, including EAML [7]. Notably, nab‐sirolimus demonstrated a 39% ORR in a phase II trial for malignant PEComa and was approved by the U.S. Food and Drug Administration for this indication [8]. Although mTOR inhibitors appear to have some efficacy in malignant EAML, the evidence remains limited, as no randomized controlled trials or large‐scale studies have been conducted.

Regarding ICI therapy, only one case of malignant EAML has reported a clinical response [5]. Lattanzi et al. described a patient with metastatic EAML who achieved a durable near‐complete response to nivolumab after disease progression on everolimus [5]. In both our case and the previously reported case, high PD‐L1 expression and CD8+ T‐cell infiltration were observed, suggesting a possible correlation between these biomarkers and responsiveness to ICIs in malignant EAML.

Furthermore, four cases of malignant PEComa responsive to ICIs have been reported [1, 9, 10]. McBridge et al. described a metastatic PEComa with high PD‐L1 expression that achieved a complete response (CR) with pembrolizumab [10]. Testa et al. reported two PEComa cases that responded to ICI therapy: one with high TMB (17 Muts/Mb), and the other treated with concurrent radiotherapy [9]. Djerroudi et al. described a case of metastatic PEComa associated with Lynch syndrome that was MSI‐high with a TMB of 21 Muts/Mb and achieved CR with pembrolizumab [1]. These biomarkers are predictors of ICI efficacy in various malignancies [11]. Although these findings suggest that known biomarkers may help identify patients likely to benefit from ICIs for malignant PEComas, further data collection is required to support these preliminary observations.

In addition to *TSC1/2* mutations, numerous studies have identified *TFE3* gene rearrangements in subsets of PEComas, including EAMLs [4, 9, 12, 13]. Previous studies have reported that TFE3 expression is observed on IHC in 29%–31% of PEComas, and *TFE3* gene rearrangements are present in 14% of cases [12, 13]. *TFE3*‐rearranged PEComas exhibit distinct clinicopathological features from conventional PEComas [12, 14]. Histologically, they lack spindle cells or fat components and predominantly consist of epithelioid cells with a clear or eosinophilic cytoplasm arranged in nests. These tumors typically lack the expression of muscle markers, such as SMA or desmin, show diffuse HMB‐45 positivity with weak or absent Melan A expression, and are usually not associated with *TSC1/2* mutations, rendering mTOR inhibitors less effective. Moreover, these patients tend to display a more aggressive clinical course [9, 12, 14].

In the present case, nuclear TFE3 positivity was noted on IHC, but *TFE3* gene rearrangement was not detected by FISH. Nevertheless, several clinicopathological features such as nested architecture, SMA negativity, absence of *TSC1/2* mutations, and aggressive behavior were consistent with *TFE3*‐rearranged PEComa. Discordance between IHC and FISH findings has been previously reported [4, 12]; however, the clinical significance of TFE3 positivity on IHC in the absence of gene rearrangement remains uncertain.

## 4 Conclusion

We report a rare case of malignant EAML in which durable disease control was achieved using nivolumab. EAMLs with high PD‐L1 expression and CD8+ T‐cell infiltration may respond to ICI therapy. Therefore, the evaluation of PD‐L1 and CD8 expression by IHC may be useful in guiding systemic treatment strategies for malignant EAML.

## Ethics Statement

The study was approved by an institutional review board (approval number: M2019‐172).

## Consent

The patient provided informed consent.

## Conflicts of Interest

The authors declare no conflicts of interest.

## Data Availability

The data that support the findings of this study are available from the corresponding author upon reasonable request.
